# The effect of distraction on change detection in crowded acoustic scenes

**DOI:** 10.1016/j.heares.2016.08.015

**Published:** 2016-11

**Authors:** Theofilos Petsas, Jemma Harrison, Makio Kashino, Shigeto Furukawa, Maria Chait

**Affiliations:** a UCL Ear Institute, 332 Gray's Inn Rd, London, UK; b Human Information Science Laboratory, NTT Communication Science Laboratories, NTT Corporation, 3-1, Morinosato-Wakamiya, Atsugi-shi, Kanagawa, Japan

## Abstract

In this series of behavioural experiments we investigated the effect of distraction on the maintenance of acoustic scene information in short-term memory. Stimuli are artificial acoustic ‘scenes’ composed of several (up to twelve) concurrent tone-pip streams (‘sources’). A gap (1000 ms) is inserted partway through the ‘scene’; Changes in the form of an appearance of a new source or disappearance of an existing source, occur after the gap in 50% of the trials. Listeners were instructed to monitor the unfolding ‘soundscapes’ for these events. Distraction was measured by presenting distractor stimuli during the gap. Experiments 1 and 2 used a dual task design where listeners were required to perform a task with varying attentional demands (‘High Demand’ vs. ‘Low Demand’) on brief auditory (Experiment 1a) or visual (Experiment 1b) signals presented during the gap. Experiments 2 and 3 required participants to ignore distractor sounds and focus on the change detection task. Our results demonstrate that the maintenance of scene information in short-term memory is influenced by the availability of attentional and/or processing resources during the gap, and that this dependence appears to be modality specific. We also show that these processes are susceptible to bottom up driven distraction even in situations when the distractors are not novel, but occur on each trial. Change detection performance is systematically linked with the, independently determined, perceptual salience of the distractor sound. The findings also demonstrate that the present task may be a useful objective means for determining relative perceptual salience.

## Highlights

• Distraction is measured by presenting distractor stimuli during a scene gap.
• Scene maintenance in memory depends on availability of resources during the gap.
• This dependence appears to be modality specific.
• Scene maintenance also prone to bottom up distraction even when distractors not novel.
• Performance depends on the perceptual salience of the distractor sound.

A key issue in our pursuit to understand listening in crowded environments involves uncovering how perceptual encoding of the auditory scene is affected by interference from distracting events or from listeners’ competing perceptual goals. Distraction reflects a basic function of the perceptual system - a mechanism that enables potentially relevant events, outside of the current focus of attention, to penetrate perception (Lavie, 2010, Parmentier, 2014). The key questions relate to what determines which unattended sounds will capture attention, and how the process of attentional capture affects the perceptual representation of other elements in the scene (Stothart et al., 2015, Mentis et al., 2016).

In hearing, distraction has often been studied using paradigms which involve embedding task relevant and task irrelevant sound features within a single sound stream and measuring the extent to which performance (usually quantified by response time; RT) is affected by irrelevant feature changes (e.g. Schröger and Wolff, 1998, Schröger et al., 2000, Roeber et al., 2003, Horváth et al., 2011, Boll and Berti, 2009; for a review see Dalton and Hughes, 2014, Frings et al., 2014).

Here we study distraction in the context of a change detection paradigm. Change detection is a core capacity of hearing (Demany et al., 2010, Cervantes Constantino et al., 2012). The auditory system is widely assumed to serve as an ‘early warning system’ which continuously scans the unfolding acoustic scene for behaviourally-relevant events (e.g. those that could indicate the approach of predators or prey) even when attention is focused elsewhere. A change detection task is therefore a pertinent and ecologically relevant means by which to probe listeners' susceptibility to distraction and its effects on auditory scene analysis.

**Fig. 1:**
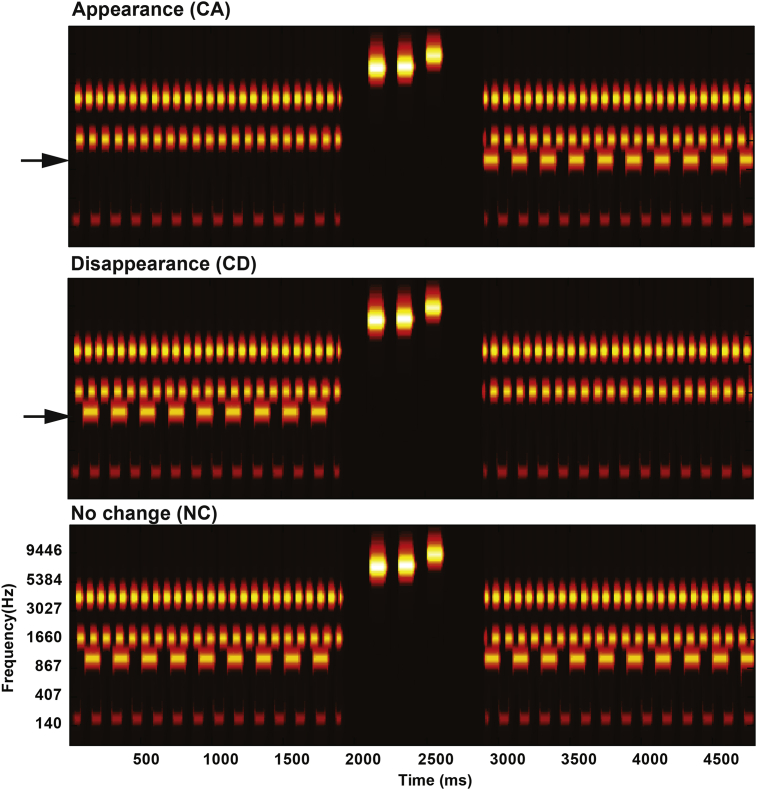
Examples of ‘scene’ stimuli. Shown are the three variations (‘Change appear’, ‘Change disappear’, and ‘No change’; changing sources are indicated by arrows) of a scene with 4 sources. They were then presented in random order during the experiment, blocked by change type (NC and CA or NC and CD) and distraction condition (as detailed for each experiment). In these examples the silent gaps, inserted partway through the scene, are filled with a tone triplet as used in Experiment 1a and Experiment 2. The plots represent ‘auditory’ spectrograms, generated with a filter bank of 1/ERB wide channels equally spaced on a scale of ERB-rate. Channels are smoothed to obtain a temporal resolution similar to the Equivalent Rectangular Duration.

Stimuli are artificial acoustic ‘scenes’ composed of several (up to twelve) concurrent sound-sources (auditory objects), each consisting of a sequence of tone pips characterized by a unique frequency and rate. Listeners are instructed to monitor the unfolding ‘soundscapes’ for occasional changes manifested as the appearance or disappearance of a source. In a previous series of experiments (Cervantes Constantino et al., 2012), we demonstrated that these stimuli are perceived as a composite ‘sound-scape’ in which individual streams can be perceptually segregated and selectively attended to, and are therefore good models for the challenges encountered in natural listening situations. This paradigm has been extensively used in our laboratory for probing the ability to detect changes in crowded acoustic environments (Cervantes Constantino et al., 2012, Sohoglu and Chait, 2016; Southwell et al., in press). Here we use a variant of the basic stimulus that incorporates a 1000 ms silent gap inserted partway through the scene, with changes (50% of the trials) occurring immediately thereafter (See Fig. 1). Distraction is quantified by measuring change detection performance as a function of the properties of an interfering signal presented during the gap. The behavioural relevance of the signal is varied across experiments.

The gap models scene interruptions which listeners regularly experience in the environment. Such disturbances may occur as a result of energetic masking, acoustic occlusion, or movement of the listener. To maintain continuity of our perceptual experience, the auditory system must rely on a memory store which retains scene information over short durations (Demany et al., 2010). The susceptibility of these mechanisms to distraction and processing load is at the centre of the present work. Presumably, to detect the scene changes, listeners must encode and preserve the pre-gap scene in short-term memory and compare this representation to the signals presented after the interruption (see also Nolden et al., 2013a, Nolden et al., 2013b). Stimuli presented during the gap may disrupt this process by capturing attention away from the maintained signal (Zimmermann et al., 2016, Parmentier, 2014) or by depleting the computational resources required for maintenance. To uncover these effects, the experiments below measured how change detection performance is affected by limited availability of attentional resources during the gap or by the presence of a (task irrelevant) distractor.

Our results demonstrate that the maintenance of scene information in short-term memory is affected by the (un) availability of attentional resources during the gap, and that this dependence appears to be modality specific. We also show that these processes are susceptible to bottom-up driven distraction even in situations when the distractors are not novel, but occur regularly on each trial. The extent of the disruption is systematically linked with the, independently determined, perceptual salience of the distractor.

## 1 General methods

### 1.1 Stimuli

Fig. 1 presents an example of the ‘acoustic scene’ stimuli used in this series of experiments. The stimulus was originally developed for the work that preceded this study (Cervantes Constantino et al., 2012) and the reader is referred to that paper for detailed information about the artificial acoustic scene signals. In brief, stimuli were artificial ‘scenes’ populated by multiple (4 or 12) streams of pure-tones designed to model sound sources. Each source is characterized by a different carrier frequency (drawn from a pool of fixed values spaced at 2*ERB between 100 and 4846 Hz; Moore and Glasberg, 1983), and is furthermore amplitude modulated (AM) by a square wave – such that the source can be seen as a sequence of tone pips. Source AM rates were randomly drawn from a pool of 15 fixed values between 3 and 35 Hz (random phase). This pattern mimics the temporal structure of many natural sounds. The amplitude of each source was fixed independently of the number of sources in a scene. Therefore, as is the case in natural environments, increasing scene size was associated with increased overall scene loudness. Scenes were 3000–5000 ms in duration including a 1000 ms silent gap inserted partway through the scene (between 1000 and 2000 ms after onset; The scene signal was ramped off and on before and after gap offset, respectively). The two scene parts (pre- and post-silent gap) were either identical (referred to as ‘no change’ stimuli; NC) or differed such that a new source appeared after the silent gap (‘change appear’; CA) or an existing source disappeared after the silent interval (‘change disappear’; CD). The set of carrier frequencies and modulation patterns was chosen randomly for each scene. To enable a controlled comparison between conditions, NC, CA and CD stimuli were generated as triplets sharing the same carrier frequencies and modulation patterns (but differing by the appearance or disappearance of a source; see Fig. 1). They were then presented in random order during the experiment, blocked by change type (NC + CA or NC + CD) and condition (as described for each experiment below). Each block contained equal numbers of no change (NC) or change (CA or CD) scenes such that the occurrence of change was unpredictable.

Stimuli were synthesized with a sampling rate of 44,100 Hz and shaped with a 30 ms raised cosine onset and offset ramp. They were presented with an EDIROL UA-4FX sound card (Roland Corporation) over headphones (Sennheiser HD 555) at a comfortable listening level (∼60–70 dB SPL), self-adjusted by each participant. Stimulus presentation was controlled using the *Cogent* software (http://www.vislab.ucl.ac.uk/cogent.php).

### 1.2 Procedure

The experiments were conducted in an acoustically-shielded booth (IAC, Winchester, UK). Experimental sessions lasted about 2 h and consisted of a practice session with feedback, followed by the main experiment without feedback, divided into runs of approximately 10 min each. Subjects were instructed to fixate at a cross presented in the centre of the display, and perform a change detection task whereby they pressed a keyboard button as fast as possible when they detected a change within the scene (CA or CD). Additional task procedures are described below.

### 1.3 Analysis

Dependent measures are response time (RT; measured between gap offset and the subject's key press) and d’ score. In the event when hit rate = 1 and/or false positive rate = 0 resulting in an undefined d’, the scores were corrected by subtracting half a trial. The α level was *a priori* set to 0.05.

## 2 Experiment 1a

We first measured the extent to which the maintenance of scene information in short-term memory is dependent on the availability of general processing resources. This was investigated in the framework of a dual task design where listeners were required to perform a task with varying perceptual demands (‘High Demand’ vs. ‘Low Demand’; matched in terms of procedural structure; see below) on brief auditory signals presented during the gap. If scene maintenance draws on (central) processing resources, performance in the change detection task should be reduced under the ‘High Demand’ relative to under the ‘Low Demand’ task.

### 2.1 Methods

#### 2.1.1 Stimuli & procedure

The basic stimuli are as described in ‘general methods’, above. In this experiment the gaps contained a sequence of three 100 ms tones, with frequencies ranging between 7 and 10 kHz, presented with an inter-onset-interval of 200 ms (for a total duration of 500 ms). The frequencies of tone1 and tone2 were randomly chosen from the above range. The frequency of tone3 varied as described below. The tone sequence was introduced 200 ms after gap onset, and ended 300 ms before gap offset (See Fig. 1). The interval between triplet offset and the onset of the second part of the scene was set at a duration sufficiently long to eliminate attentional blink effects (AB). AB refers to the inability to successfully identify the second of two sensory targets presented in rapid succession (Duncan et al., 1997, Shen and Mondor, 2006, Horváth and Burgyán, 2011).

**Fig. 2:**
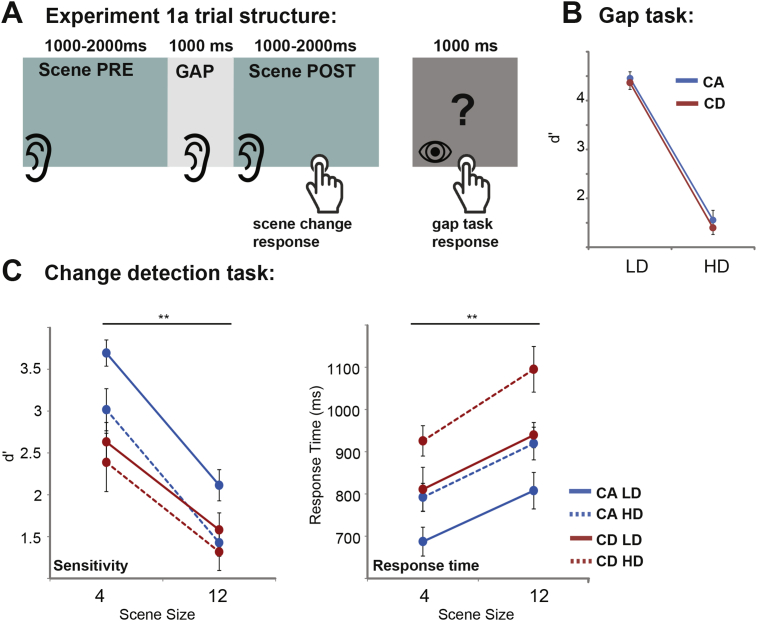
Stimuli and results for Experiment 1a: **A**. Trial structure: trials began with a presentation of an acoustic scene which was interrupted by a silent gap after 1000–2000 ms. Participants then attended to the tone triplet stimuli, presented during the gap and performed a task as instructed. After a period of 1000 ms, the acoustic scene resumed and subjects were directed to press a button as quickly as possible if they detect a change (appearance or disappearance of a source). After scene offset a question mark appeared on the screen and participants indicated their response to the gap task at that time. **B**. Performance in the gap task **C**. Results of the change detection task. Error bars here and elsewhere plot SEMs. The results demonstrate worsened change detection performance (both in terms of d’ and RT) under the HD relative to the LD, gap task.

In the high demand (HD) condition, tone1 and tone3 were identical (50% of the trials) or differed by ± 1500 Hz (this value was set based on pilot experiments). Subjects were instructed to respond when tone1 and tone3 were identical. In the low demand (LD) task, triplets had a similar structure except that in half of the trials tone2 and tone3 were merged into a single, 300 ms long pure tone. Participants were instructed to detect these instances. The overall trial structure (Fig. 2A) consisted of a presentation of the pre-change (PRE) scene, followed by the gap during which subjects performed the LD or HD task (in separate blocks) but withheld the response. Participants then listened to the second part of the scene (POST) and indicated, by pressing a button as soon as possible, when a change (the appearance of a new source, or disappearance of a previously present source) had occurred. After scene offset, a question mark appeared on the screen for 1000 ms and subjects indicated their response to the gap task at this time. Therefore the HD and LD tasks were procedurally identical with the only difference pertaining to the computational demands incurred by the two tasks during the gap. The HD task required participants to encode and maintain frequency information, whilst the LD task involved detecting a clear pattern difference, and did not require memorizing triplet specific information. Pilot experiments demonstrated that this indeed had a measurable effect on performance (see ‘results’ below). Importantly the frequency of the triplet tones was set far above the range occupied by scene sources such that any interference between the change detection and gap task is not attributable to simple energetic masking. The level of the tones was set to 6 dB above that of the scene. During the practice session, subjects were instructed to adjust the overall volume of the signal (scene + gap tones) such that it was at a comfortable level. Experiment 1a ran in four separate blocks (CA LD, CA HD, CD LD, CD HD) which were presented in random order. Participants were instructed to prioritize the gap task and guess the occurrence of change if not sure.

#### 2.1.2 Participants

Data from eleven paid participants (mean age 24.4 years; 6 female) are reported. The data from 5 additional subjects were excluded from the analysis because their performance on the gap task indicated a failure to prioritize the gap task over the change task (see below). All participants reported normal hearing and no history of neurological disorders. Experimental procedures (here and in subsequent experiments) were approved by the research ethics committee of University College London, and written informed consent was obtained from each participant.

### 2.2 Results

#### 2.2.1 Gap task

Since subjects were instructed to prioritize the gap task, we expected performance on this task to be independent of the change detection task - i.e. not affected by whether it was presented within a (easier) CA or (harder) CD trial. Subjects whose gap task (LD or HD) performance within CA vs CD blocks differed by more than Δd’ = 1 were therefore excluded from further analysis. Five participants were omitted in this way. Gap task performance for the remaining participants is plotted in Fig. 2B. A Repeated measures ANOVA with load and change type as factors showed a strong main effect of load (F(1,11) = 216.89; *p* < *0.001*) and confirmed no effect of change type (F(1,11) = 1,64; *p* = *0*.229). The lack of difference between CA and CD in the LD condition may be attributable to ceiling effects; Importantly there was no difference between the two conditions in the HD task, where performance on both conditions is well below ceiling (paired sample *t*-test: t = 1.596 p = 0.142).

Under the assumption that participants were equally engaged by the LD and HD tasks, this pattern of results confirms that the gap task successfully manipulated load. The lack of main effects or interactions with change type suggests that the remaining subjects were indeed prioritizing the gap task over the change detection task.

#### 2.2.2 Change detection task

Fig. 2C depicts change detection performance as a function of gap task. A repeated measures ANOVA on d’ scores with load (LD vs HD), change type (CA vs CD), and scene size (4 and 12) as factors revealed main effects of load (F(1,10) = 18.94; *p* = *0.001*), change type (F(1,10) = *27,61; p* < *0.001)* and scene size (F(1,10) = 151,55; *p* < *0.001*), with no interactions. Main effects of change type (CA > CD) and scene size are commonly observed in change detection experiments (see Cervantes Constantino et al., 2012, Sohoglu and Chait, 2016). These reflect the perceptual advantage for appearing vs. disappearing sources and that change detection performance generally deteriorates as the scene becomes more crowded. Importantly, the main effect of load demonstrates that performance on both CA and CD trials was affected by the demands of the competing (gap) task such that change detection performance decreased under high load relative to low load.

The response time analysis demonstrated a similar pattern: Main effects of load (F(1,10) = 23.83; *p* = *0.001*), change type F(1,10) = 31.24; *p* < *0.001*) and scene size (F(1,10) = 34.13; *p* < *0.001*) – confirming that the effects of load are reflected in increased response times as well as decreased sensitivity to change.

While very similar, the stimuli in the HD and LD tasks were not exactly identical: In the LD condition, the gap signals sometimes consisted of two, rather than three, sound events (see methods). To address the concern that this difference might have affected the results we repeated the analysis by including only the subset of LD trials where listeners heard 3 tones (exactly identical to those in the HD condition). The results (not shown) demonstrated an identical pattern to that reported above.

Thus increased load deleteriously affected change detection performance despite the fact that the gap signals and scene signals did not overlap spectrally. The reduction in performance could be a consequence of exhaustion of resources by the HD gap task, which led to impaired maintenance of the scene during the retention period and therefore to failure to compare the representation of the pre-gap scene to the post-gap scene. Another possibility is that after the HD gap task, listeners were slower at re-orienting attention towards the onset of the post-gap scene. The substantially slowed down (about 100 ms) response time in the HD condition is indeed consistent with that interpretation. However the marked decrease in sensitivity (reduction in d’) between LD and HD conditions is in line with impaired maintenance of scene information in short-term memory, suggesting that both effects may have contributed to the observed failure in change detection performance.

## 3 Experiment 1b

**Fig. 3:**
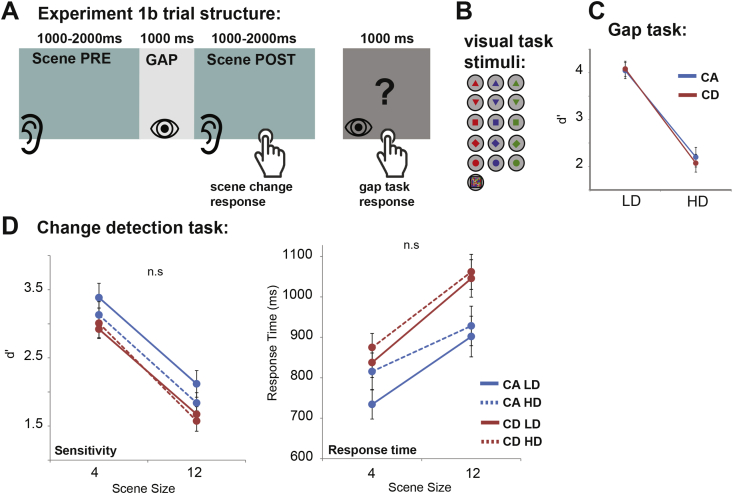
Stimuli and results for Experiment 1b: **A**. Trial structure: trials began with a presentation of an acoustic scene which was interrupted by a silent gap after 1000–2000 ms. Participants then attended to visual stimuli, presented during the gap and performed a task as instructed. After a period of 1000 ms, the acoustic scene resumed and subjects were directed to press a button as quickly as possible if they detect a change (appearance or disappearance of an object). After scene offset a question mark appeared on the screen and participants indicated their response to the gap task at that time. **B**. The visual shapes used in the gap task. **C**. Performance in the gap task **D**. Results of the change detection task. The results demonstrate no effect of gap task load on change detection performance (both in terms of d’ and RT).

In experiment 1b we repeated the same general paradigm but using a visual-based gap task. The task, requiring participants to monitor a series of rapidly presented visual shapes, mirrors common ways in which we deploy visual attention (e.g. reading) and is therefore a relevant task with which to investigate how visual task demands might interfere with auditory processing. Importantly, the task was designed to be procedurally similar to the auditory task in experiment 1a (see Fig. 3A) to allow for a direct comparison between performance in the two experiments.

### 3.1 Methods

#### 3.1.1 Stimuli & procedure

Stimuli and procedures were identical to those in Experiment 1a, except that the gap task was based on visual signals (rapid serial visual presentation; RSVP). The visual stimuli were rapid random sequences of simple geometric shapes. We used five different shapes (circle, square, triangle, upside-down triangle and diamond) drawn in one of three colours (red, green or blue) as well as a visual checker pattern, generated by multiple random-sized ellipses that were each drawn in a random colour (red, green, or blue), and at a random position within the target square (see Fig. 3B). Stimuli were presented in the centre of a grey screen (RGB: 190,190,190) at a distance of about 52 cm from the subject's eyes. The simple shapes were presented at a visual angle of 7.4°. The checker pattern was larger (13.7°). Random sequences of these shapes were generated anew for each trial. Sequence duration was 800 ms and contained 5 or 6 serially presented shapes (the number was determined for each subject individually based on performance in the practice session). In the high demand (HD) task, subjects were instructed to remember the first stimulus (exact combination of shape and colour) in the sequence and determine whether it was presented again, later in the sequence. In the low demand task, subjects were to respond if the checker pattern stimulus was present within the sequence. This stimulus was physically very different from the rest and was easily detected within the stimulus stream. In contrast, the HD task required participants to perform an attentionally demanding feature conjunction search (Treisman, 1982) throughout the length of the sequence.

#### 3.1.2 Participants

Data from Seventeen paid subjects are reported (mean age 24 years; 8 female). All subjects confirmed normal hearing and no history of neurological disorders. Using the same criteria as described for Experiment 1a, above, one additional participant was excluded due to failure to prioritize the gap task over the change detection task (Δd’>1). Eight of the participants had also participated in Experiment 1a.

### 3.2 Results

#### 3.2.1 Gap task

Fig. 3C shows the average performance (d’) in the gap task. Overlapping traces for CA and CD demonstrate that the type of change (CA vs CD) did not affect performance. A repeated measures ANOVA on d’ data with load and change type as factors, confirmed that the effect of change type was not significant (F(1,16) = *0.62; p* = *0.443*). As in Experiment 1a, above, the lack of difference between CA and CD in the LD condition may be attributable to ceiling effects; Crucially, there was no difference between the two conditions in the HD task, where performance on both is well below ceiling (paired sample *t*-test: t = 1.305 p = 0.21).

We note that, despite similar performance in the piloting stages, d’ in the visual HD task was somewhat higher than that observed for the auditory HD task in Experiment 1a (mean d’ here was 2.1; while in Experiment 1a d’ = 1.5). Importantly, a strong main effect of load was established (F(1,16) = 73.65; *p* < *0.001*), confirming that the task successfully manipulated processing demands.

#### 3.2.2 Change detection task

Fig. 3D plots change detection performance as a function of gap task. A repeated measures ANOVA on d’ scores with load, change type, and scene size as factors revealed main effects of change type (F(1,16) = 14.45; *p* = *0.002*) and scene size (F(1,16) = 158.12; *p* < *0.001*) only, with no interactions. The effect of load, including interactions involving this factor, was not significant (p > 0.1), suggesting that the load of the visual gap task did not affect change detection performance.

The response time analysis revealed a similar pattern: Main effects of change type (F(1,16) = 51.43; *p* < *0.001*) and scene size (F(1,16) = 30.465; *p* < *0.001*), with no interactions. The effect of load was not statistically significant (*p* = *0.355*) indicating that the perceptual demands of the gap task did not affect the response time to appearing or disappearing sources.

#### 3.2.3 Comparison across modalities

The effects of the auditory and visual gap tasks on change detection were compared directly using a repeated measures ANOVA with load, change type and scene size as within subject factors and modality (auditory or visual) as a between-subjects factor. The d’ based analysis revealed main effects of load (F(1,26) = 21.3; *p* < *0.001*), change type (F(1,26) = 42.9; *p* < *0.001*) and scene size (F(1,26) = 285.1; *p* < *0.001*) as well as interactions between modality and load (F(1,26) = 6.4; *p* = *0.018*), change type and load (F(1,26) = 5.9, *p* = *0.022*; due to there being a larger load-related reduction in performance on CA relative to CD stimuli) and change type by scene size by modality (F(1,26) = 4.5 *p* = *0.043*). The significant interaction between modality and load confirms that the visual and auditory gap tasks indeed had qualitatively different effects on performance. The RT based test revealed comparable results: main effects of load (F(1,26) = 13.9; *p* = *0.001*), change type (F(1,26) = 81.1; *p* < *0.001*) and scene size (F(1,26) = 55.5; *p* < *0.001*) as well as an interaction between modality and load (F(1,26) = 5.8; *p* = *0.023*).

In both d’ and RT analyses there was no main effect of modality (p > 0.4) suggesting an overall similar level of performance on the change detection task (when collapsed over change type, scene size, and load), irrespective of the nature of the competing task (auditory or visual). However the interaction between modality and load, observed in both the d’ and RT analyses demonstrates that while the auditory HD gap task significantly impaired change detection performance, the visual HD gap task did not affect change detection.

## 4 Experiment 2

The paradigm for Experiment 2 was identical to that in Experiment 1a, except participants were instructed to ignore the gap sounds and focus on the change detection task only. In half of the trials (separately blocked) the gaps were empty (no distractors) and in the other half the gaps were filled with triplets identical to those in Experiment 1a. We sought to understand whether task irrelevant sounds (distractors), positioned well away from the spectral range occupied by the auditory scenes, would impact on change detection performance.

### 4.1 Methods

#### 4.1.1 Stimuli

Scene stimuli were as described in ‘general methods’, above. The silent interval within each scene was either (equal probability) empty or contained a tone triplet identical to that used in experiment 1a (HD task). Scenes contained 4 or 12 sources and were blocked according to Change type (CA or CD) and Gap (silent gap or a gap with a triplet). This resulted in four main blocks, which were delivered in random order (counter balanced across subjects; CA silent, CA triplet, CD silent and CD triplet). During the experiment the subjects were instructed to look at a cross on the centre of the screen, ignore the triplets and focus on the change detection task.

#### 4.1.2 Subjects

10 paid subjects participated in this experiment (7 female; mean age 24.5 years). All reported normal hearing and no history of neurological disorders. Eight of the participants had also participated in Experiment 1, above. However, this experiment was conducted before Experiment 1, all of the participants were therefore naïve to the triplets.

### 4.2 Results

**Fig. 4:**
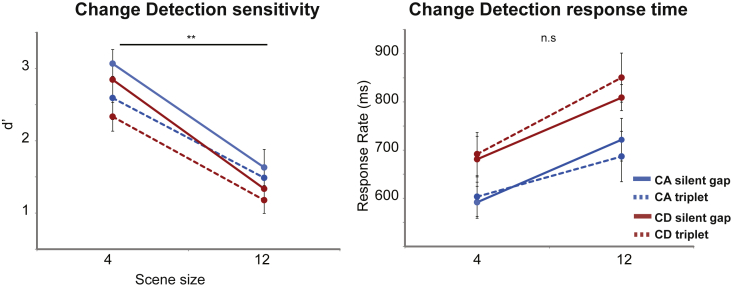
Results for Experiment 2. Change detection as measured by d’ was significantly reduced when the gaps contained a triplet relative to when they were empty. Response times did not differ between conditions.

The results of Experiment 2 (d’ sensitivity scores and response times) are presented in Fig. 4. A repeated measures ANOVA on d’ scores confirmed significant main effects of change type (F = 7.97, *p* = *0.02*) scene size (F = 122.36, *p* < *0.001*) and gap (F = 13.28, *p* = *0.005*). There were no factor interactions. These results suggest that the presence of the triplet interferes with the memory maintenance of scene information in a manner that is detrimental to performance. This effect was present in the detection data only: A repeated measures ANOVA on RT showed significant main effects of change type (F = 14.80, *p* = *0.004*) and scene size (F = 32.70, *p* < *0.001*) but the effect of gap was not significant (F = 0.18, *p* = *0.683*). There were no interactions.

Overall the results demonstrate that the addition of the tone triplets caused a significant reduction in change detection behaviour notwithstanding the fact that they were irrelevant to the task, and that subjects were instructed to ignore them. Since the triplets occupied a spectral range distant from that of the scene stimuli, the effect is attributable to distraction (‘informational masking’) rather than any physical interaction between the stimuli in the gap and the scene sources. The triplet signals likely inadvertently attracted resources away in a manner that interfered with the representation of the acoustic scene in memory during the gap. Subjects were apparently unable to resist this distraction despite the fact that the stimulus wasn't novel (similar triplets were presented on each trial) and its timing was predictable (see also, Chait et al., 2010).

## 5 Experiment 3

Experiment 3 builds on the results of Experiment 2 to measure whether the observed distraction effects are reflective of the subjective salience of the distractor. Towards this aim, we used environmental sounds which were ranked for salience, and measured whether a signal's subjectively-measured salience correlates with the extent to which it interferes with listeners' change detection capacity.

### 5.1 Methods

#### 5.1.1 Stimuli

**Fig. 5:**
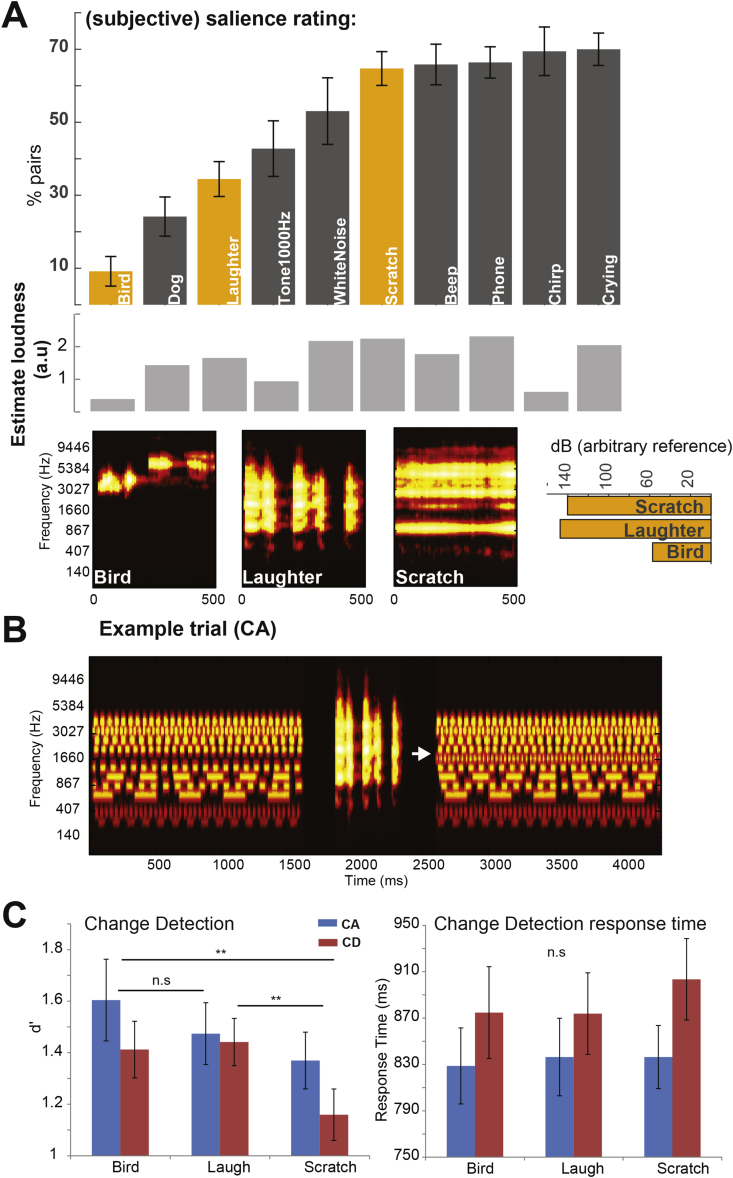
Stimuli and results of Experiment 3. **A**. top: Results of the salience rating experiment. The 10 sounds are arranged based on the % of pairs in which they were indicated as ‘more salient’. The signals selected for the main experiment are indicated in orange. Middle: The output of an ERB-based loudness model. Bottom: Spectrograms of the three selected sounds and their overall energy. **B**. An example trial (CA; gap sound = ‘Laughter’). The changing source is indicated with an arrow. **C**. Results of the change detection task. Change detection as measured by d’ was affected by the type of sound in the gap. Response times did not differ between conditions. (For interpretation of the references to colour in this figure legend, the reader is referred to the web version of this article.)

The general paradigm was similar to that in Experiment 2, with the following differences: (a) The scene size was fixed at 12 sources; (b) Instead of triplets of pure tones, the gaps contained complex sounds (as described below). These had a duration of 500 ms and were inserted in the middle of the gap (between 250 and 750 ms after gap onset). An example of a CA trial is shown in Fig. 5B.

Scene stimuli were blocked according to change type (CA vs. CD). The gap sounds varied randomly (counterbalanced across trials). Subjects were instructed to ignore the gap sounds and focus on the change detection task.

#### 5.1.2 Salience scale

We used a set of 10, equal duration (500 ms) sounds from the study by Liao et al. (2016). At a first stage (before the main experiment, and using a separate group of participants), we estimated the sounds' relative subjective salience. Signals were grouped in pairs (every possible combination). On each trail, a pair of sounds was presented sequentially with an inter-sound interval of 200 ms. Participants were directed to indicate which of the two sounds was more ‘salient’. Pairs were presented in random order, with each pair occurring 4 times within the session. The order of sounds within a pair was counter balanced across presentations.

To derive a relative salience scale, sounds were sorted according to the number of times they were selected as “more salient” across all pairwise comparisons (See Fig. 5A). The resulting ratings are similar to those obtained in Japan by Liao et al. (2016). Outputs of an ERB-based loudness model are given below (light grey bars). A loudness measure was produced by a model in which the acoustic signal was filtered with a bank of bandpass filters of width 1 ERB (Moore and Glasberg, 1983) with centre frequencies spaced 1/2 ERB from 30 Hz to 16 kHz. The instantaneous power of each filter output was smoothed with a 20 ms window and elevated to power 0.3 to approximate specific loudness (Hartmann, 1998). Outputs were then averaged across channels. This model was preceded by a combination of high-pass and low-pass filters to approximate effects of outer and middle ear filtering (Killion, 1978).

Three sounds (orange bars) - from the lower, mid and upper band of the subjective salience scale (“bird”, “scratch” & “laughter”) were selected for the main experiment. Fig. 5A shows the time frequency representation and energy (sum of squares) of the signals. It is clear that the employed sounds possess different spectro-temporal characteristics and Liao et al. (2016) indeed demonstrate that these are related to subjective salience. Importantly, ‘Laughter’ and ‘Scratch’ were selected because they span a comparable spectral range and contain similar overall energy. While the loudness model output didn't always match well with subjective salience ratings, the three selected sounds did exhibit increased perceptual salience with increased loudness (see also Liao et al., 2016).

#### 5.1.3 Subjects

Data from 10 subjects (mean age 27.5 years; 3 female) were used to derive the salience scale. Nineteen (different) paid subjects participated in the main experiment (mean age 28.1 years; 12 female). None of the participants had participated in any of the previous experiments. One additional subject was excluded from the final dataset due to very low performance (false positive rates over 2 STDE above the mean for the group). All subjects reported normal hearing and no known neurological disorders.

### 5.2 Results

Fig. 5C summarises the change detection performance (d’ and RT scores). A repeated measures ANOVA on d’ scores with change type (CA vs CD) and distractor (bird, laughter, scratch) as factors revealed a main effect of distractor (F(2,36) = 6.75; *p* = *0.003*) only, with no interactions. The lack of an effect of change type, robustly observed in the previous experiments, is likely due to a combination of noise and the use of scene size 12 stimuli only, where these effects are smaller.

Post-Hoc pairwise comparisons (Bonferroni corrected) between the different distractor conditions revealed a significant difference between “bird” and “scratch” (*p* = *0.019*) as well as between “laughter” and “scratch (*p* = *0.016*).

The same analysis, applied to RT data, revealed a main effect on change type only (F(1,18) = 6.81; *p* = *0.01*). Thus, similarly to Experiment 2, above, the effect of salience was confined to d’.

That we observe a difference between ‘bird’ and ‘scratch’ is perhaps not surprising given the large differences in overall energy between the two sounds (see also, Liao et al., 2016). However, it is remarkable that ‘laughter’ and ‘scratch’ had significantly different effects on change detection sensitivity despite their similar spectral and energy characteristics (see Fig. 5A). This association between subjective salience and distraction demonstrates that the maintenance of auditory information (in this case, pertaining to scene contents) can be systematically impaired by sounds of varying perceptual significance: Despite the fact that the sounds were equally irrelevant to the task, some were more distracting than others in a manner that correlated to independently obtained subjective salience reports. Overall the findings are consistent with the conclusions in Experiment 2 that the maintenance of scene information is vulnerable to interference, in a bottom-up driven manner, from irrelevant, explicitly ignored, co-occurring events.

## 6 Discussion

In this series of experiments we demonstrated that the maintenance of scene information in the context of a change detection task depends on the availability of resources that are shared with other perceptual processes (Experiment 1). We also show that this maintenance is vulnerable to the presence of irrelevant acoustic events (Experiment 2, 3) even when these occur on each trial and are completely expected. We hypothesize that this occurs because attention is involuntarily drawn to these events.

### 6.1 The effect of load and distraction on change detection

Firstly, it is important to note that, despite the various manipulations of distraction and despite the 1000 ms silent gap preceding the scene change, change detection performance remained reasonably high even in the crowded scenes used here (12 independent sources). This speaks against the general notion of ‘change deafness' (Eramudugolla et al., 2005). Instead the data are a testament of the auditory system's sensitivity to changes in our surroundings (Demany et al., 2010, Cervantes Constantino et al., 2012) and to its ability to resist a variety of scene disruptions.

A common finding in most of the present experiments is the consistent advantage of CA vs CD changes. Under most conditions, listeners tend to respond faster and more accurately to appearance relative to disappearance events. This effect is explored at length in Cervantes Constantino et al. (2012; see also Pavani and Turatto, 2008, Sohoglu and Chait, 2016). The CA advantage may stem from low level differences between the two conditions such as the reliance of CA on adaptation effects and the known differences between onset and offset responses in the auditory cortex (Scholl et al., 2010, Phillips et al., 2002; see also Andreou et al., 2015; but these should have reduced effects here due to the long gaps). It is also possible that the CA benefit stems from an inherent bias towards appearance (Cole and Kuhn, 2010, Franconeri et al., 2005). Indeed, the fact that the advantage persists after 1000 ms of silence could be taken as evidence for such a tendency to perceptually prioritize appearing objects.

### 6.2 Effect of load

The results of Experiment 1a demonstrate that depletion of resources during the gap had detrimental effects on change detection. The HD and LD tasks were matched in terms of the nature of the stimuli and general task procedures and only differed in their demands on resources. The observed effect is therefore attributable to an influence of load on the representation of the scene in memory during the gap.

Early investigations of the effect of task demands on auditory memory have yielded mixed results. Many studies concluded auditory memory to be generally independent of task demands (e.g. Cowan et al., 1990, Keller and Cowan, 1994, Demany et al., 2001, Kaernbach and Schlemmer, 2008). For example, Demany et al. (2001) failed to find effects of attention in a delayed matching experiment. However, Keller et al. (1995) reported reduced performance in a pitch memory task when a distractor task was presented during the retention period, suggesting that active maintenance is susceptible to interference by distractors. Similarly, Green and McKeown (2007), using a probe-signal paradigm, demonstrated that the memory trace of the cue can be affected by active maintenance.

Importantly, in the present paradigm participants were not instructed to memorize the scenes. The task was explained to the subjects as a change detection task. Our results do not speak to whether this maintenance is accomplished in an automatic echoic memory store or via an active process (Payne and Sekuler, 2014, Jiang et al., 2015), but demonstrate that whatever this representation may be, it is dependent on the availability of computational resources.

Experiment 1b investigated the modality specificity of these effects by repeating the experiment with a visual load task that was matched procedurally to that in Experiment 1a. The issue of whether different modalities draw on separate, independent resource pools or are shared across a single, central pool accessible to all sensory modalities has been a central question in cognitive neuroscience (e.g. Duncan et al., 1997, Larsen et al., 2003, Alais et al., 2006, Kreitz et al., 2015) that has yielded mixed findings (Dyson et al., 2005, Muller-Gass et al., 2005, Talsma et al., 2006, Rees et al., 2001, Sculthorpe et al., 2008, Restuccia et al., 2005, Otten et al., 2000, Müller et al., 2002, Parks et al., 2011, Chait et al., 2012, Salmela et al., 2014, Raveh and Lavie, 2015, Molloy et al., 2015, Masutomi et al., 2016). For example, Rees et al., 1997, Rees et al., 2001 showed that visual load, but not auditory load, modulates processing of a task-irrelevant visual motion stimulus. Conversely, Molloy et al. (2015) recently demonstrated that high visual load led to reduced early responses in auditory cortex to irrelevant pure tones.

The results of Experiment 1b suggest that the maintenance of scene information in the context of a change detection task draws on modality specific resources – a visual gap task resulted in no effects of load despite being procedurally matched to the auditory task and roughly equated for difficulty.

However, the conclusion about modality specificity must be taken with some caution. Despite our efforts to match the auditory and visual tasks, the results indicated that the visual task was marginally easier than the auditory task (in Experiment 1b the HD d’ was 2.1; while in Experiment 1a d’ = 1.5). More broadly, it is difficult to exclude the possibility that other visual tasks might elicit a different set of results.

### 6.3 Salience and distraction

Experiments 2 and 3 demonstrate that although the task-irrelevant gap sounds occurred on each trial, and were therefore expected (Volosin and Horváth, 2014, Parmentier et al., 2011, Chait et al., 2010), participants were not able to resist distraction. This was especially surprising in Experiment 3 where an effect of salience was observed regardless of the fact that we used only 3 different sounds which were presented repeatedly throughout the 1.5 h long experimental session. Whilst distraction by novel events (singletons) is perhaps not surprising - the brain must allow unexpected, potentially relevant, events to penetrate perception (Lavie, 2010, Parmentier, 2014), we demonstrate that completely expected task irrelevant stimuli also induce a cost to perception.

In vision, accumulating research is uncovering the low level saliency maps that define the aspects of the scene to which attention is drawn in a bottom up driven manner (e.g. Peters et al., 2005, Koene and Zhaoping, 2007, Itti and Koch, 2001) and the object categories which possess perceptual primacy (Jenkins et al., 2003, Ro et al., 2007, New et al., 2007). Similar efforts are emerging in the auditory modality (Kayser et al., 2005, Kaya and Elhilali, 2014, Liao et al., 2016, Neuhoff, 1998). It is clear that loudness is a major factor in determining salience (Liao et al., 2016), but there is also evidence that salience is affected by a sound's emotional valance (e.g. Arnal et al., 2015, Max et al., 2015) and context (Li et al., 2013, Horváth et al., 2011, Parmentier et al., 2011, Röer et al., 2014a). Understanding the various feature dimensions that contribute to a sound's perceptual prominence is imperative for designing alarms, human computer interfaces and for controlling distractibility in the increasingly sound polluted environments in which we routinely operate (Mentis et al., 2016; Stothart et al., 2015, Röer et al., 2014b).

Even though the sounds we used in Experiment 3 were selected arbitrarily and likely do not capture the full range of salience of environmental sounds, the evident correspondence between subjective and objective assessment of salience is compelling: The demonstration that subjectively-rated salience is correlated with measures of ‘acoustic prominence’ as quantified by distractibility, marks the present approach as a useful paradigm for determining salience in an objective, and ecologically pertinent manner. This method may also be a relevant means for assessing distractibility in the context of individual differences or clinical populations (Sörqvist and Rönnberg, 2014, Dinica et al., 2015, Forster and Lavie, 2015).
